# CASE REPORT Penetrating Injury of the Orbit by a Needlefish

**Published:** 2013-08-06

**Authors:** Miho Ohtsubo, Kenya Fujita, Kazuhiro Tsunekawa, Shunsuke Yuzuriha, Kiyoshi Matsuo

**Affiliations:** ^a^Department of Plastic and Reconstructive Surgery, Shinshu University School of Medicine, Matsumoto; ^b^Department of Plastic and Reconstructive surgery, Nagano Children's Hospital, Azumino, Japan

## Abstract

**Objective:** We present a very rare case of penetrating injury into the orbit by a needlefish. The patient underwent extirpation twice at another hospital. **Methods:** We performed foreign body removal from a right subbrow incision under general anesthesia. **Results:** The foreign body was successfully removed and the patient's diplopia recovered gradually after surgery. **Conclusions:** Treatment was similar to that used for penetrating injury. As there is a risk of secondary infection, it is important to completely remove the fish body, followed by vigorous irrigation and debridement.

The needlefish belongs to the family Belonidae and is found in subtropical and tropical regions of all oceans.^1^ These fish are found near the water surface and attack bright flashes of light, because they are carnivorous predators that feed on small fish. Swimmers are sometimes injured by needlefish, and some authors have reported needlefish-penetration injuries, for example, in the upper jaw,^2^ chest,^3^ abdomen,^3^ and lower limbs.^4^ Here, we present our experience with penetrating injury into the orbit by a needlefish.

## CASE REPORT

A 27-year-old man was injured by a fish striking his right lower eyelid while swimming in Okinawa, a Japanese sea resort. Initially, he received treatment by an ophthalmologist at a local hospital in Okinawa. There was a small laceration in the right lower eyelid, and a foreign body was detected in the right orbit by computed tomography (CT). The foreign body was extirpated from the initial wound in his lower eyelid 2 days after the injury under local anesthesia at the local hospital. Postoperative CT revealed residual foreign body within the right orbit. Reoperation was performed from the upper conjunctival fornix incision under local retrobulbar anesthesia, but the foreign body could not be found. The patient visited our office with his treatment summary from the ophthalmologist 8 days after the injury. The patient had received prophylactic antibiotic therapy with cefaclor at a dose of 750 mg per day from the day of the injury.

**Figure 1:**
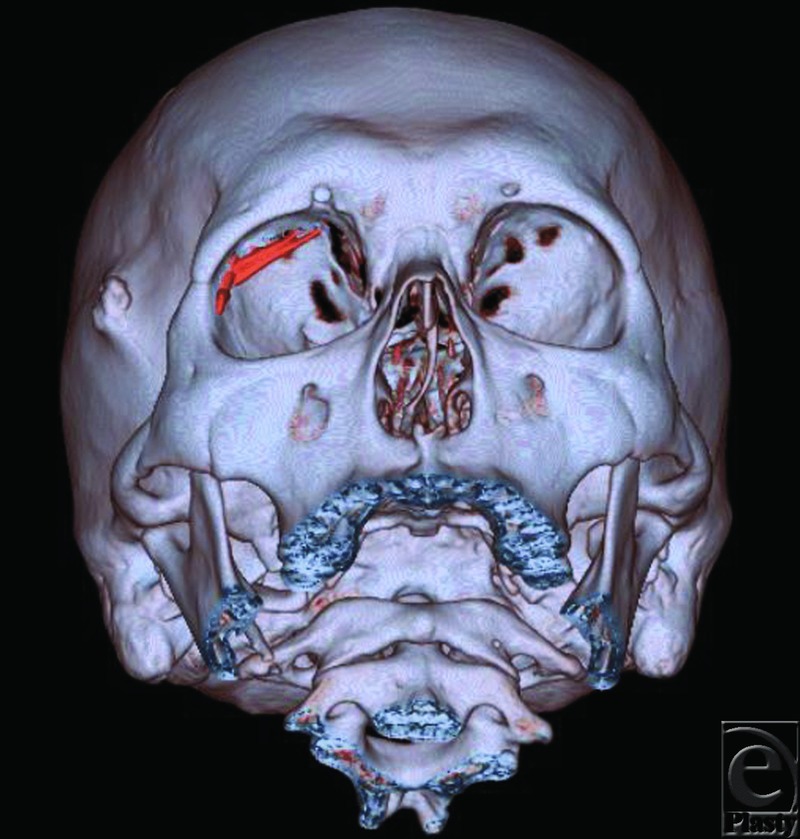
CT indicating calcifications in the upper part and outer side of the right orbit. The elongated objects depicted in red are the foreign bodies.

Initial evaluation in our office showed painful swelling and severe blepharoptosis of the right upper eyelid. The patient was carefully examined by ophthalmologists and plastic and reconstructive surgeons (the authors: M.O. and K.F.). Visual function tests indicated diplopia in the right vision and restriction of right eye abduction. Visual acuity of the right eye showed no abnormalities. His right eyelid was swollen, but there were no elevations in his serum C-reactive protein level (0.13 mg/L) or the ratio of neutrophilic leukocytes (58.8%). Computed tomographic findings showed calcifications of 25 mm in the upper part of the superior rectus muscle and 7 mm on the outer side of the lateral rectus muscle in the right orbit (Fig 1).

**Figure 2:**
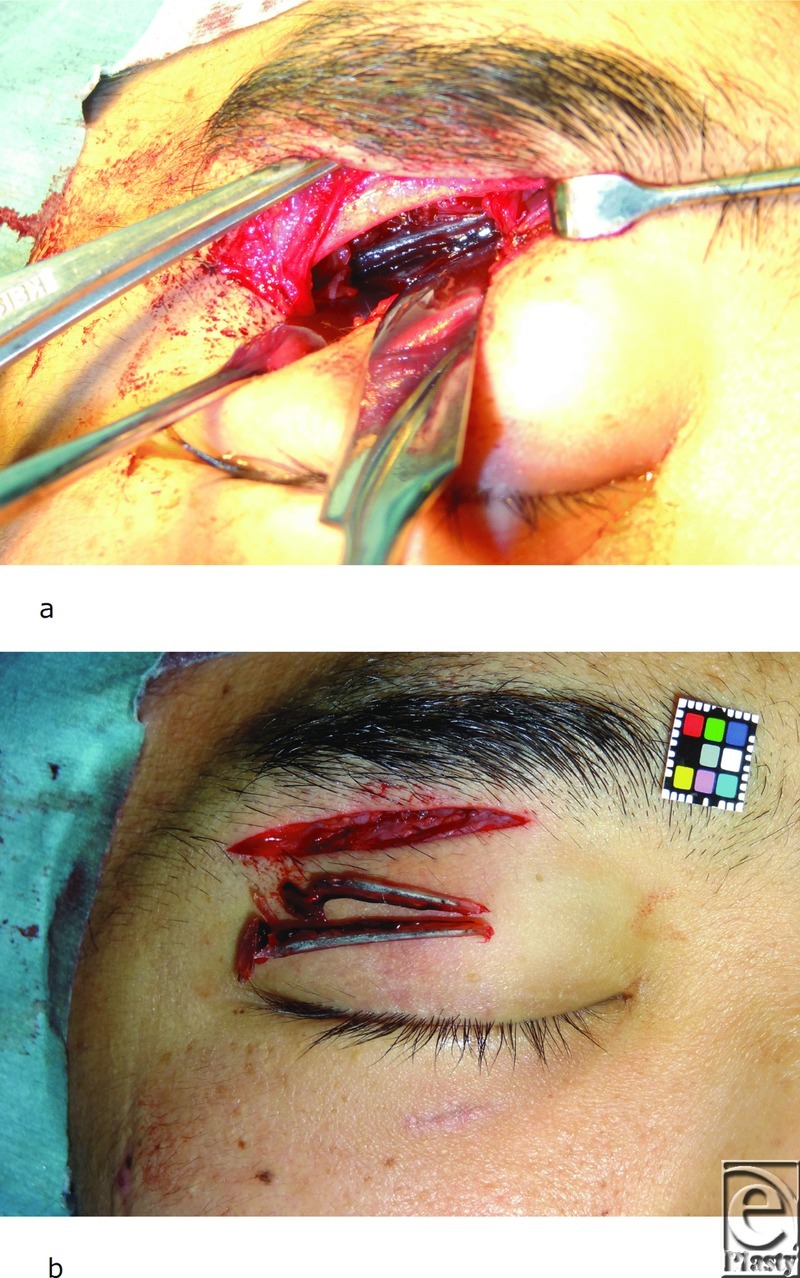
(*a*) Foreign bodies under the periosteum. (*b*) Removal of foreign bodies in the right orbit.

After admission, we performed foreign body removal from a right subbrow incision under general anesthesia 10 days after the injury. Following subperiosteal dissection, we found granulation tissue penetrating the periosteum at the upper orbital roof. We incised the periosteum from the granulation tissue toward the near side and then found the foreign bodies directly beneath the periosteum. We removed 2 elongated bodies that were suspected to be needlefish jaws (Fig 2). Postoperative CT did not reveal obvious foreign body in the right orbit. The patient received cefazolin (1 g) and clindamycin (600 mg) intravenously every 12 hours from day 10 to day 14 after the injury. Close ophthalmological follow-up was maintained for 4 months. The patient's diplopia recovered gradually after surgery and his postoperative course was uneventful.

## DISCUSSION

**Figure 3:**
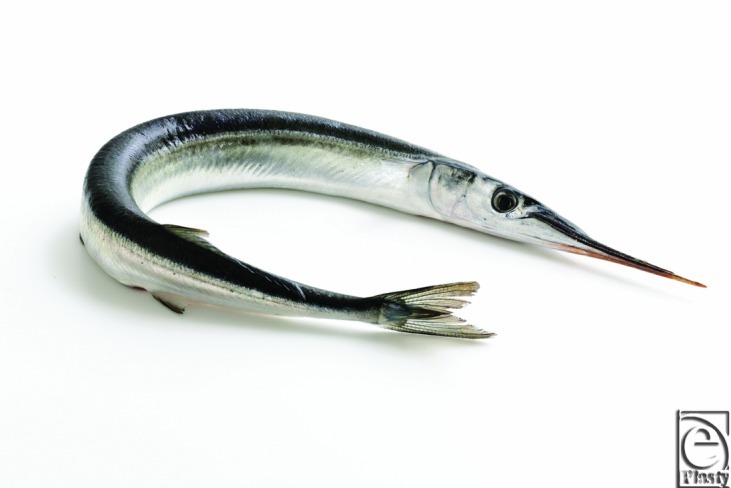
Needlefish. Photo compliments of © Fo2mas—Fotolia.com.

Penetration injury due to needlefish is a rare occurrence. The needlefish is elongate,^1^ silver in color, with a dark blue stripe. The upper and lower jaws are elongated with long beaks that have small sharp teeth (Fig 3).^2^

Needlefish injury may be confused with a stingray or catfish injury, as all 3 fish may cause stab injury. However, stingray and catfish injuries show increased severity due to envenomation, which produces sharp pain out of proportion to the degree of tissue damage. The envenomation is treated by immersion in hot water for 30 to 90 minutes until the pain improves due to inactivation of the heat-labile venom.^5^^,^^6^ Needlefish do not produce envenomation. Therefore, treatment of the injury is similar to penetrating injury, and it is determined primarily by the site of damage.

In this case, foreign bodies were trapped in the orbit from a small insertion site on the lower eyelid. The wound was small but resulted in eye movement disorder due to the residual foreign bodies in the orbit.

In this case, CT was beneficial for discovery of the foreign bodies in the orbital cavity. X-ray examination is important because it cannot penetrate foreign bodies, such as the beak and teeth of fish. With regard to injuries to the head, neck, chest, and abdomen, CT imaging may be useful in stabilized patients.^3^ Furthermore, at extirpation of the foreign body, there is no clear “end” to the needlefish projectile, so the surgeon cannot readily tell by inspection of the removed material if it has been entirely removed. Therefore, surgeons should not hesitate to repeat this scan if retained foreign body is suspected after initial surgery, as in this case.

There is a high risk of secondary infection after needlefish injury. This is particularly true for members of the Belonidae family, as they are carnivorous and their putrid dentition is a source of infection. In this case, intraorbital foreign bodies were removed 10 days after the injury. The bone had begun to undergo decomposition. Reduction of wound infection was achieved by vigorous irrigation and debridement.

Antibiotic therapy for any marine-acquired infection should include therapy against Vibrio species. *Vibrio parahaemolyticus* or *Vibrio vulnificus* should be suspected in patients with rapidly progressive cellulitis or myositis from a marine injury. The recommended antibiotics should include intravenous third-generation cephalosporins, ciprofloxacin or imipenem-cilastatin.^7^ Our patient did not suffer severe infection without use of these antibiotics.

## CONCLUSION

We presented a case of penetrating injury into the orbit by needlefish. Treatment of the injury is similar to a stab wound. Vigorous irrigation and debridement are necessary to reduce infection in the wound.
